# En–DeNet Based Segmentation and Gradational Modular Network Classification for Liver Cancer Diagnosis

**DOI:** 10.3390/biomedicines11051309

**Published:** 2023-04-28

**Authors:** Suganeshwari G, Jothi Prabha Appadurai, Balasubramanian Prabhu Kavin, Kavitha C, Wen-Cheng Lai

**Affiliations:** 1 School of Computer Science and Engineering, Vellore Institute of Technology, Chennai 600127, Tamil Nadu, India; 2 Computer Science and Engineering Department, Kakatiya Institute of Technology and Science, Warangal 506015, Telangana, India; 3 Department of Data Science and Business Systems, College of Engineering and Technology, SRM Institute of Science and Technology, SRM Nagar, Chengalpattu District, Kattankulathur 603203, Tamilnadu, India; 4 Department of Computer Science and Engineering, Sathyabama Institute of Science and Technology, Jeppiaar Nagar, Rajiv Gandhi Salai, Chennai 600119, Tamil Nadu, India; 5 Bachelor Program in Industrial Projects, National Yunlin University of Science and Technology, Douliu 640301, Taiwan; 6 Department Electronic Engineering, National Yunlin University of Science and Technology, Douliu 640301, Taiwan

**Keywords:** liver segmentation, encoder–decoder network, gradational modular network, computed tomography, cancer diagnosis

## Abstract

Liver cancer ranks as the sixth most prevalent cancer among all cancers globally. Computed tomography (CT) scanning is a non-invasive analytic imaging sensory system that provides greater insight into human structures than traditional X-rays, which are typically used to make the diagnosis. Often, the final product of a CT scan is a three-dimensional image constructed from a series of interlaced two-dimensional slices. Remember that not all slices deliver useful information for tumor detection. Recently, CT scan images of the liver and its tumors have been segmented using deep learning techniques. The primary goal of this study is to develop a deep learning-based system for automatically segmenting the liver and its tumors from CT scan pictures, and also reduce the amount of time and labor required by speeding up the process of diagnosing liver cancer. At its core, an Encoder–Decoder Network (En–DeNet) uses a deep neural network built on UNet to serve as an encoder, and a pre-trained EfficientNet to serve as a decoder. In order to improve liver segmentation, we developed specialized preprocessing techniques, such as the production of multichannel pictures, de-noising, contrast enhancement, ensemble, and the union of model predictions. Then, we proposed the Gradational modular network (GraMNet), which is a unique and estimated efficient deep learning technique. In GraMNet, smaller networks called SubNets are used to construct larger and more robust networks using a variety of alternative configurations. Only one new SubNet modules is updated for learning at each level. This helps in the optimization of the network and minimizes the amount of computational resources needed for training. The segmentation and classification performance of this study is compared to the Liver Tumor Segmentation Benchmark (LiTS) and 3D Image Rebuilding for Comparison of Algorithms Database (3DIRCADb01). By breaking down the components of deep learning, a state-of-the-art level of performance can be attained in the scenarios used in the evaluation. In comparison to more conventional deep learning architectures, the GraMNets generated here have a low computational difficulty. When associated with the benchmark study methods, the straight forward GraMNet is trained faster, consumes less memory, and processes images more rapidly.

## 1. Introduction

Liver cancer ranks sixth among all cancers in terms of frequency of occurrence. According to the latest data from the Cancer Data statement [1], it is the second leading cause of cancer-related mortality in women. In 2020, 905,700 people were diagnosed with, and 830,200 people died from liver cancer globally [2]. Liver cancer and subordinate liver cancer are its two main categories. Hepatocellular cancer accounts for 80% of all incidences of liver cancer [3]. Approximately 700,000 individuals a year globally die from hepatocellular carcinoma (HCC), making it the most common cause of cancer-related death [4]. Cirrhosis due to alcohol consumption, viruses, and fatty liver disease caused by obesity are the key risk factors related to the primary liver malignancies [5]. Ultrasound, elastography, MRI, and CT scans are just a few of the imaging techniques that can be used to identify and detect liver cancer. From the above, CT scans are a form of periodic picture test [6]. This is an X-ray-based test that can easily identify tumors; when a patient undergoes CT imaging, a thin X-ray is directed at them and rapidly spun round the body, resulting in numerous images of the identical organ or tissue. The X-ray detectors pick up the ensuing signals from the patient, and the computer analyzes the data to create slices of the body. When compared with traditional X-rays, CT scans provide clarity and more details about bones, soft tissues, internal organs and blood vessels. Since these cross-sectional views reveal more detail than standard X-rays, we call them tomographic images.

After the machine’s computer acquires a series of consecutive slices, they can be viewed singly or can be digitally layered to generate an image of the patient. CT scans allow doctors to see various internal structures, such as the bones, organs, and tissues, as well as any anomalies. Basic structures and potential lesions or anomalies are easily detected on a CT picture [7]. Digital imaging communications in medicine (DICOM) files are used to store these CT scans; they consist of a header with the patient’s information, study parameters, etc., and image datasets. Most of these images are analyzed by radiologists, and hence there are risks associated with expert fatigue, a low imaging quality, and pathologic variability. Computer-Aided Diagnosis (CAD) systems have been used as a tool in order improve the aid offered to these professionals [8]. However, the CT image segmentation of tumorous regions remains crucial due to the intensity of the similarity between the tumor and the surrounding tissues [9,10]. For this reason, the photos should be processed and enhanced in order to identify malignant tissue. Liver cancer can be detected on a CT scan by comparing it with the pixel strength found in a healthy liver and noting whether the tumor area is darker (hypodense) or brighter (hyperdense) [11].

The segmentation of CT scan pictures is a tedious and lengthy process in a clinical context for many reasons. For example, liver CT scans naturally span over 150 slices, lesion forms are often ill-defined, the contrast between lesions and the surrounding tissue may be poor, the size and shape of the liver vary from patient to patient, and the liver intensity may be comparable to that of other organs. [12,13]. With these issues in mind, scientists have developed a variety of computer-aided diagnostic techniques to separate liver tissue from malignancies in abdominal CT scans. In the past, doctors manually removed malignancies from liver scans using a variety of methods. However, the tumor was often not completely removed using these techniques. Rather than evaluating the image pixel by pixel, most of these previous techniques relied on edge detectors and were either manual or semi-automatic. Various deep learning approaches have also been utilized for fully or partially automated liver tumor segmentation. One of the most popular approaches is the use of a Convolutional Neural Network (CNN) [14]. Researchers have used CNNs and their extensions, a fully connected layer and UNet, for liver and tumor segmentation [15].

### 1.1. Contributions

In this study, we develop the first deep neural network model, or En–DeNet, to automatically separate liver cancer from two datasets of images and quantify their shape metrics so that the model can be utilized in order to categorize the liver into a binary classification. In addition, we propose GraMNet, a unique and computationally efficient deep learning strategy that can be employed in order to analyze liver images using CNNs. As a solution, we employ network components called SubNets, each of which can produce high-impact features. To keep the computational complexity to a minimum, we start with tiny compact SubNet modules. Second, we employ sequential incremental construction techniques in order to construct the networks in series and parallel configurations. This allows the construction to be virtually any type of specialized network. The key to this method is locking in the parameters by using only one small SubNet throughout training. The original network is expanded in either dimension, and then a new SubNet is created and trained independently. To obtain the best possible network performance, we take baby steps in this direction. The sum of layers, kernel size, series increase, and parallel network are all options that can be freely selected using our method.

### 1.2. Paper Organization

This section of the paper is prearranged as follows. Section 2 delivers an analysis of the relevant literature for the diagnosis of liver cancer. Section 3 provides an impression of the datasets that were analyzed, as well as the segmentation and classification methods that were employed. The outcomes of the experiments are discusses in Section 4. To conclude, in Section 5, we provide some analysis and final thoughts.

## 2. Related Works

The two-step process proposed by Saha Roy et al. [16] for identifying liver tumors involves first performing mask R-CNN and then conducting MSER tumor identification. The categorization was completed with the help of a deep learning-based HCNN, While the segmentation framework attempts to separate healthy tissue from malignant liver tissue, the classification technique calculates a multi-class categorization of the tumor. The purpose of this research was to identify an approach that could recognize tumors without the human influence. Our proposed technique achieves a near-peak segmentation and classification performance, exhibiting the uppermost precision for lesion diagnosis and a high recall value. In most cases, our technique accurately identifies liver tumors as either HCC or cysts. The model achieved a classification accuracy of approximately 94%. This work applies a mask R-CNN-based process for liver component segmentation and a MSER-based methodology to classify hepatic multitudes.

The deep learning-based innovative process for segmenting and categorizing liver tumors was recently adopted by Balasubramanian et al. [17]. Specifically, the model they created makes use of three steps in order to realize this aim, namely (a) pre-processing, (b) liver segmentation, and (c) classification. In the pre-processing phase, the received Computed Tomography (CT) images are equalized via a histogram in order to boost the contrast and filter the image through a median filter to lessen the noise. The liver is subsequently extracted from the CT abdominal picture by applying a refined mask region-based segmentation model (Mask R-CNN). To avoid overfitting, an Enhanced Swin Transformer Network is applied to the segmented picture using adversarial propagation (APESTNet). The trial results show that the proposed approach works well, is not easily affected by noise, and outperforms the baseline of an extensive variety of CT images.

Hence, Shukla et al. [18] proposed a method for the automatic detection of liver tumors and lesions in abdominal MRI images by utilizing 3D affine invariant and form parameterization techniques. Counterpoint parameterization establishes a consistent model that can be applied to generate superficial images of the organ during the modelling procedure, which helps to eliminate the common problems associated with concave surfaces. As a first step, the analysis method is employed to isolate the liver of the body. Then, the training procedures are carried out using a Cascaded Fully Convolutional Network (CFCN), where the input region allows for the error rate to be minimized. The findings and validity are determined via the stage analysis of datasets that include both training and testing images. When applied to the analysis of liver tumors, a CFCN achieves an accuracy of 94.21%, while taking less than 90 s to calculate the results per volume.

The problems encountered by Peng et al. [19] in the tumor classification process were as follows: (1) the huge variety in the tumor size and in the texture and grayscale; and (2) the inaccurate segmentation of liver tumor boundaries due to blurred boundaries between the tumor and adjacent organs. Tumor UNet++ extracts the liver tumor from the source CT images. Through the process of learning to recognize tumor edges, the suggested segmentation approach is able to address the issue of segmentation error at tumor edges. Finally, to prevent the deep network from becoming overly sensitive or overfit, the images are adaptively cropped; this is based on the volume of the tumor. A newer version of Dense Block is utilized thoroughly for the minute differences observed in tumors that are either benign or malignant. Finally, the network’s retrieved features are added to the patient’s tumor volume, sex, and age before being passed to a classifier. The HD95 and dice cutoffs for liver tumor segmentation were found to be 12.1 mm and 71.9%, respectively. The classification findings show that the area under the curve is 87.5%, the accuracy is 82.4%, the specificity is 79.8%, and the sensitivity is 84.4%. The results for both the segmentation and classification are superior to those obtained using conventional approaches and networks.

To fully automate the end-to-end segmentation process, Li et al. [20] created a deep supervision network for segmentation that combines channel attention with Res-UNet++ (ECA residual UNet++). As a starting point, this article uses the UNet++ framework. By using a context-aware residual block feature encoder, we can improve feature extraction and avoid the degradation of deep networks. By replacing the cross-entropy loss function with Dice Loss, we can optimize network parameters in a way that takes into the account both the map and the spatial context that are used. According to the Liver Tumor Segmentation Benchmark LITS dataset, the liver accuracy is 95.8% and the liver tumor segmentation accuracy is 89.3%. This paper’s results reveal that compared to previous algorithms, this method provides a good segmentation performance (93%), making it a useful reference for achieving the fine segmentation of the liver and liver cancers in computer-assisted diagnosis and therapy.

Using an improved hybrid deep learning model, Shaheen et al. [21] present an automated cirrhosis liver disease classification. In this investigation, MRI (Magnetic Resonance Imaging) is one of the options being investigated. In the first stage, via the input of MRI pictures, the noise is reduced using an (EGF). The tumor is extracted from the image using binomial thresholding. Finally, the Gray-Level Co-occurrence Matrix (GLCM) and (GRLM) are used. Hence, in order to classify cirrhosis liver illness, Convolutional Neural Network and Capsule Network are integrated to form a hybrid model called HCNN-CN. In addition an optimization strategy is employed in order to adjust the neural network’s parameters. When compared to other methods, the suggested HCNN-CN-AEPO shows an accuracy and sensitivity of 98.6%, respectively, on the real-time dataset. The trial results verify the accuracy of the suggested HCNN-CN-AEPO in the diagnosis of tumors.

To improve the CT pictures, Khan et al. [22] present a multi-level GAN. This involves the computer-assisted diagnosis of liver cancer via the use of generated improved images. We employ qualitative and measurable analysis, including performance measures and computer-aided diagnostics, to demonstrate its application to three publicly available datasets. This metric analysis achieves a mean structure resemblance index of 0.45 and a maximum SNR of 16.20 dB. The projected multi-level GAN successfully generates improved biomedical images with conserved organizational details and a favorable reduction in the artefacts, as demonstrated by the ability of AlexNet to perform binary classification. 

The existing models [16,17] use either masked R-CNN or enhanced R-CNN for segmentation, whereas other models [18,19,20,21,22] describe the use of U-Net for classification. However, the proposed model uses the encoder–decoder efficient network) for segmentation and a modular classifier for the final process. Moreover, most of the existing techniques consider only one dataset for validation, but here, two datasets are used for comparison; thus, the proposed model has a lower level of computation complexity since it has a smaller number of blocks. 

## 3. Proposed System

In this section, the proposed model for liver tumor segmentation and classification is explained. Initially, two datasets are used, which is explained in the next section. 

### 3.1. Description of Materials Datasets

**Figure 1:**
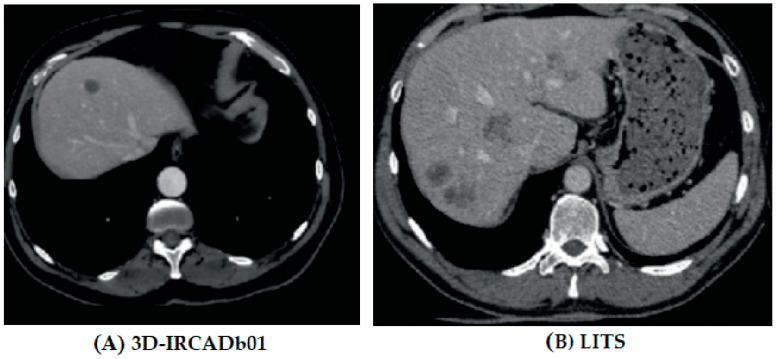
Sample images of the two datasets.

This paper’s liver and liver cancer segmentation process was trained and tested on images from two publicly obtainable datasets: 3DIRCADb01 [23] and LITS Challenge [24]. The 3DIRCADb01 dataset was challenging to utilize since it includes a high variety of data, and liver and tumors are complex. Table 1 includes specifics regarding the two segmentation datasets. Figure 1 shows the sample images of the dataset, where first figure shows the image for 3DIRCADb01 and second figures shows the sample image for LITS

### 3.2. Data Preparation

The developed method required training and testing with images acquired from the two datasets. There were seven subfolders in the 3D-ircadb01 dataset for each patient’s data retraining, which were built upon the biological position of the tumor masks. These subfolders, which included all of the tumor masks, were spread across multiple folders and thus needed to be combined into a single one. This is because the focus was on the segmentation outcome and not the tumor’s functional site.

In addition, the LITS dataset features three-dimensional images without a dedicated liver tumor mask. The data had to be transformed into two dimensions (2D) in order to work with the established algorithm. There also had be a mask set aside for the liver and the tumor. The ImageJ tool was introduced in order to prepare these data. No patient data regarding liver and no tumor masks were eliminated. The foreground was further reduced by excluding, from each patient’s data, images or slices that were taken at the beginning and conclusion of scanning when there was no liver information. Table 2 includes the total number of training and testing images; in total, 2738 images were data augmented in this research work.

### 3.3. Image Pre-Processing

The images were 512 × 512 in dimension after data augmentation. It was difficult to use these images due to limited GPU memory. Therefore, all images were resized with a factor of 0.25. In addition, the images were also normalized to have a value between 0 and 1.

Additional annotations exist within these datasets, including the segmentations of bones, vasculature, intraparenchymal tumors, and other organs by radiologists. To harmonize these data, we grouped and utilized only the radiologist annotations by demarcating the contours of the liver and ignoring the additional sub annotations of the tumors, including intrahepatic tumors, vasculature, or other abdominal organs. Specifically, we did not retain any annotations that denoted the contours of the gallbladder, the intra parenchymal vasculature, the external biliary system, the hepatic portal vein, the proper hepatic artery, or the hepatic veins. These images were stored in these databases as Digital Imaging and Communications in Medicine (DICOM) [25].

### 3.4. Architecture of En–DeNet and Network Training

**Figure 2:**
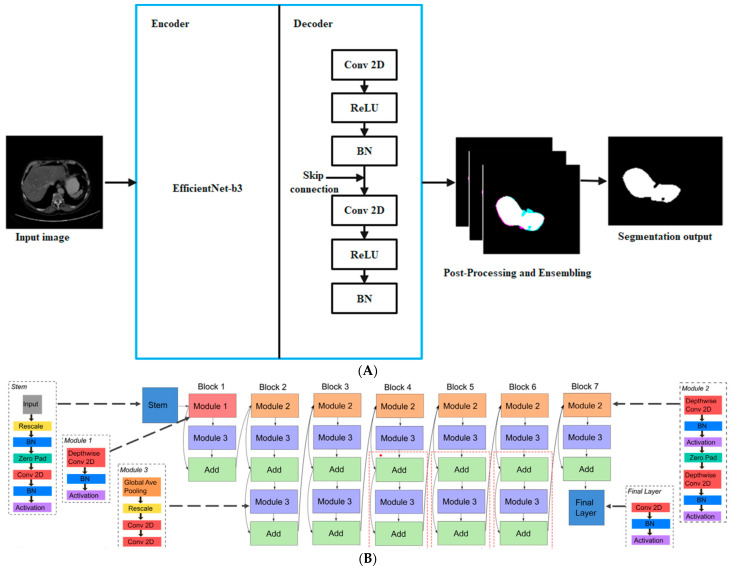
Overview of the architecture of En–DeNet. (**A**) Architecture of proposed segmentation; (**B**) Architecture of EfficientNet-b3.

The En-structure of DeNet was derived from the widely used UNet, and its two main components were the encoder and decoder (En–DeNet). The liver’s multi-level characteristics were extracted by the decoder, then these features were used to create the segmentation output. We used the classification network EfficientNet-b341 with a depth of 5 as the encoder in the En–DeNet, as the role of the encoder is vital to the good performance of the segmentation process. In contrast, the En-decoder DeNet was made up of 5 blocks, each of which consisted of 2 convolution layers with a 3 × 3 kernel. The channel counts for these clusters were (256, 128, 64, 32, 16). In addition, we used transfer learning in the En–DeNet by pre-training the EfficientNet-b3 using ImageNet42 in order to obtain rapid convergence throughout the training process. Figure 2 presents the architecture of the proposed model. 

After processing, the image of the live was cut into five sections. Four folds were used to train and validate the En–DeNet, while the fifth set was aside as test data. Photos from the same patient could be included in both sets of liver images, as the images were divided randomly between the two. Using these four sets of images, we conducted a 10-fold cross-validation, in which nine sets were used for training after enhancement and one set was utilized for validation. Then, 63% percent of the original dataset was used for training, 17% for validation, and 20% for testing. To boost the training efficiency, the number of original training photos was increased to 63 × 32 = 2016.

The binary cross entropy and Dice Loss were used to define the loss function, as follows:

(1) Loss=BCE+α×Dice,

where BCE and Dice are defined as follows:

(2) $$\mathrm{BCE}=\sum_{n}\left[y_{n}.\;lnx_{n}+\left(1-y_{n}\right)\times ln\left(1-x_{n}\right)\right]$$

(3) $$\mathrm{DICE}=\frac{2\left|X\cap Y\right|}{\left|X\right|+\left|Y\right|}$$

Here, *X* and *Y* signify the model’s prediction and the goal, respectively; $x_{n}$ and $y_{n}$ are pixel values in *X* and *Y*; and α is adjusted to 0.2 so that BCE and Dice contribute equally to the loss. The segmentation results were not improved; therefore, we included the Hausdorff distance, which is often used to incorporate the contour difference of two shapes into the loss function.

Our optimization procedure used Adam, with parameters that included a learning rate of 0.001 and a weight decay of 10^−8^. When the validation loss remained unchanged for five training epochs, the learning rate was abridged to one-tenth of its initial value using a Plateau scheduler to help the En–DeNet converge. The training was expected to take 200 epochs, although the new Plateau scheduler could conclude it sooner. Then, 16-person batches were processed at a resolution of 512 by 512 by 2 (two channels were enhanced liver images and a perfusion map).

#### 3.4.1. Binarization Post-Processing

In the last layer of En–DeNet, the sigmoid start function was used, and as a result, the pixel values of the output images fell within the range (0, 1). These pixel values were transformed into a binary form, with 1 representing pixels belonging to liver tumors and 0 representing all other regions (not tumor). In this research, the segmented images were binarized using a 0.5 threshold.

#### 3.4.2. Clearing

**Figure 3:**
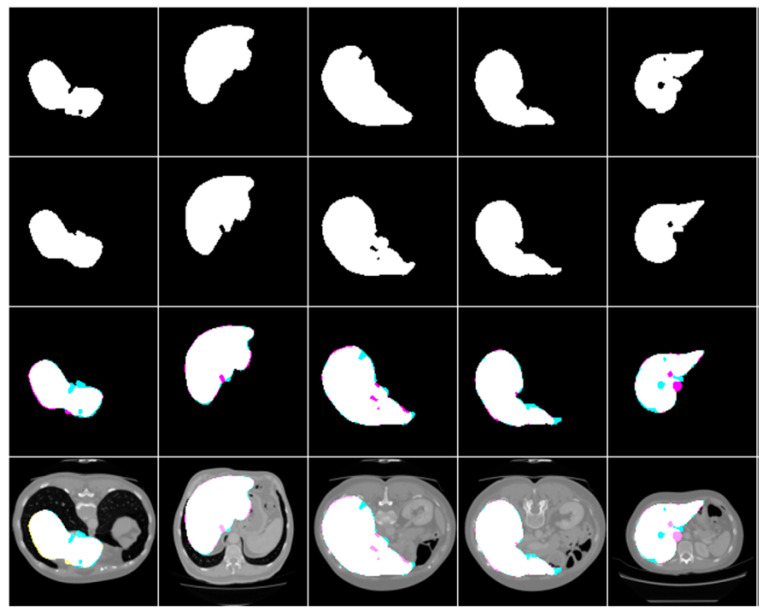
Simple liver segmentation outcomes, true Images, and overlays.

**Figure 4:**
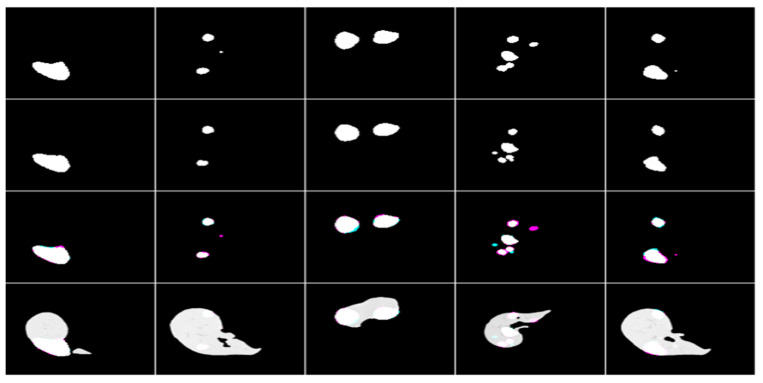
The following are examples of liver tumor segmentation together with their corresponding masks and overlap photos.

**Figure 5:**
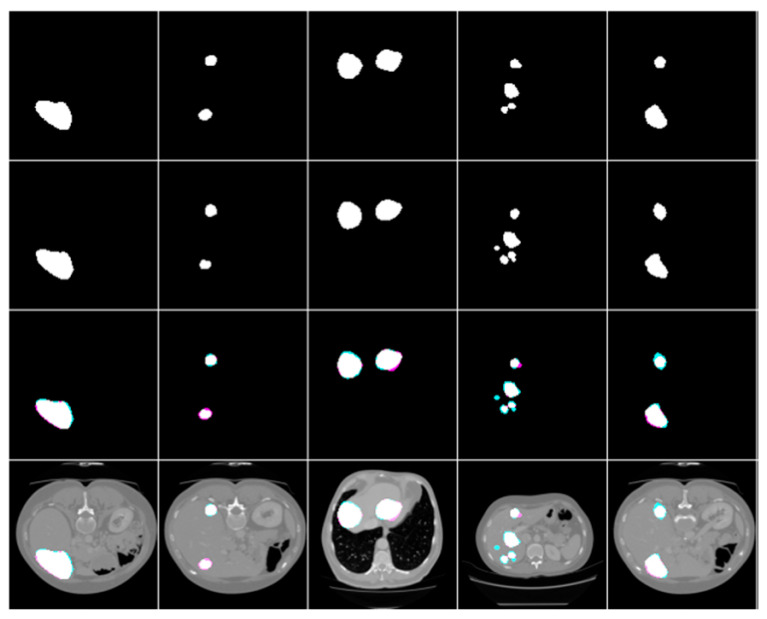
The end result of using masks and overlap pictures to segment a tumor from abdominal CT scans.

Hence, some segmented photos had tiny pieces that were incorrectly identified as malignancy. Therefore, we eliminated the fragments from the segmented images unless their combined area was less than 1024 pixels. The results of the segmentation of a sample liver and tumor are shown in Figure 3, Figure 4 and Figure 5.

### 3.5. Classification Methods

The GraMNet approach used in this study is detailed in this section. We will start with a quick summary. We next proceed to describe the inner workings of each SubNet and their interdependence. The GraMNet architecture that was actually employed in our experiment is presented below. In this final session, we will describe how we trained our network.

#### 3.5.1. Overview

The GraMNet strategy was modelled on a child’s play item, i.e., building blocks. We suggest that CNN constructions can be built using modular mechanisms in the same way that a child might use building blocks to erect a fort. Training each module was simple and straightforward, which enabled a hypothetically large network without the possible prohibitive computational expense of training the last network immediately. To achieve its goal of producing complementary information, the suggested GraMNet used a novel hybrid learning technique that positively merged various SubNets.

Our strategy consisted of serially or concurrently adding new SubNet modules on top of the pre-existing architecture. SubNet modules were superimposed on top of the existing GraMNet feature computation layers. As a result, the categorization layers were relocated to the end of the network.

In addition, the current learnable parameters of GraMNet were frozen, and only the SubNets were modified. This greatly reduces the computational burden of the back-propagation updates, especially for large networks. It is also possible to grow the network simultaneously at various points throughout the GraMNet procedure. The classification layers were moved to the end, and the back-propagation learning process was updated with the new Subnet. The present GraMNet feature maps needed to be concatenated with the SubNet feature maps; to do this, a new layer of operation was required. In the channel dimension, we joined these feature maps together.

#### 3.5.2. SubNet Architecture

**Figure 6:**
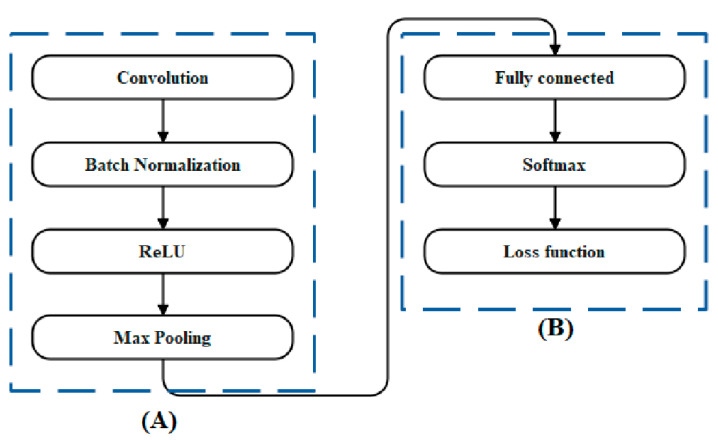
SubNet architecture. (**A**) depicts the layer groupings that made the feature-generating SubNets. (**B**) illustrates the classification layers.

Figure 6 depicts the various SubNet topologies that were taken into account for this article. Figure 6A depicts the layer groupings that made the feature-generating SubNets.

Figure 6B illustrates the classification layers. Figure 6A depicts the convolutional layer architecture, which consists of a batch normalization layer. Convolution filters can vary in both number and size across layers. The loss function for classification uses cross entropy and there is only one completely linked layer.

The output of a SubNet constructed from the L 1-layer groups, such as those in Figure 6A, and then shadowed by an L’th layer, such as that in Figure 6B, is formally defined below. In order to get started, let us define what a single input data sample is:

(4) $$X=\left\{X_{1},X_{2},\ldots ,X_{N}\right\}$$

where $X_{n}\in R^{H\times W\times D}$ is the n’th paradigm from the minibatch. Possible multi-channel images with H rows, W pillars, and D channels are represented by these inputs. The classification problem, in which there are M possible categories, is dealt with. The truth for each example is denoted by $y_{n}={\left[y_{n,1},\;y_{n,2},\;.\;.\;.\;,\;y_{n,M}\right]}^{T}\;\in R^{M}$, for n=1, 2, . . . , N.

Let us use the n-th example and $X_{n}$ as the network’s Layer 1 input. In lexicographic notation, we can write this as $x_{n}^{0}$, HWD×1 vector. Take note that this is a column vector produced by redesigning the 3D data cube in $X_{n}$. Each group of convolutional layers in Figure 6A has an output that can be written as follows:

(5) $$x_{n}^{1}=g\left(w^{l}x_{n}^{l-1}+b_{l}\right)$$

within the minibatch, for n=1, 2, . . . , N. The weight matrix $W^{1}$ represents the weights of all kernels in layer group l. $W^{1}$ has the dimensions $HWN_{f}^{1}\times HWD$, where $N_{f}^{1}$ is the total sum of filters in layer group l=1. By using max pooling layers, the dimensions can be decreased in consecutive layer groups. The vector $b^{l}$ is used to store the bias terms. By considering the vector, $x_{n}^{1}$ represents the lexicographic output of the 3D feature map cube of the present layer l.

By combining the stacked function representations of the ReLU and max pooling layers shown in Figure 6A, the following is expressed:

(6) $$g\left(x\right)=MaxPool\left(Max\left(0,x\right)\right)$$

The ReLU operation is the sum of the highest values of all elements and 0. To avoid the slowdown in learning and performance caused by vanishing gradients in other activation functions, the ReLU activation function g() is utilized in this case. The feature maps are downsampled by a factor of dimension for each channel using the *MaxPool*() operator’s 22 spatial sub-sampling kernel.

In this case, we follow the convolution layer groups with the classification layer group, as indicated in Figure 6B. In the fully connected layer, each input and output is linked by the weight matrix, and the size is equivalent to the sum of classes, M. Instead of using convolution kernels, it uses something else. Additionally, neither ReLU nor maximum pooling is used. In a fully combined layer, the function can be written as follows:

(7) $$X_{n}^{L}=W^{L}x_{n}^{L-1}+b^{L}$$

where $x_{n}^{L}={\left[x_{n,1}^{L},\;x_{n,2}^{L},\;.\;.\;.\;x_{n,M}^{L}\right]}^{T}$ is the output. The final feature map from the L−1 convolution layer is the vector $x^{L-1}$. A complete layer’s worth of biases can be found in the vector notational notation $b^{L}$. The so-called soft-max process, which standardizes the yield, comes into play after the completely connected layer.

(8) $${\hat{y}}_{n}={\left[{\hat{y}}_{n,1},{\hat{y}}_{n,2},\ldots .,{\hat{y}}_{n,M}\right]}^{T}=Softmax\left(x_{n}^{L}\right)$$

where

(9) $${\hat{y}}_{n,m}=\frac{e^{x_{n,m}^{L}}}{\sum_{j=1}^{M}e^{x_{n,j}^{L}}}$$

Note that the productivities of the SoftMax process, ${\hat{y}}_{n,m}$, are in the variety [0, 1], and

(10) $$\sum_{m=1}^{M}{\hat{y}}_{n,m}=1$$

The above-mentioned mathematical specifics can be condensed into the following:

(11) $$\hat{y}=f\left(X,\phi \right)$$

where $\hat{y}={\left[{\hat{y}}_{1},{\hat{y}}_{2},\ldots ,{\hat{y}}_{N}\right]}^{T}$ is all of the learnable parameters, and the predicted labels are provided:

(12) $$\phi =\left\{w^{l},b^{l}|l\in \left\{1,2,\ldots ,L\right\}\right\}$$

Thus, $f\left(.\right)$ represents the entire SubNet predictor module, and f stands for the network’s trainable parameters. Each minibatch’s empirical risk is used to inform an update to the learnable structures. The error function in cross entropy that is obtained empirically is as follows:

(13) $$R_{emp}\left(X,\phi \right)=-\frac{1}{M}\sum_{n=1}^{N}\sum_{m=1}^{M}y_{n,m}\times 1n\left({\hat{y}}_{n,m}\right)$$

The minibatch data X and the observable structures are the required inputs for $R_{emp}\left(\cdot \right)$. The learnable parameters in ϕ are the minibatch size represented by N, and the number of classes represented by M. Our model’s truth labels are represented by the mutable $y_{n,m}$, whereas the predicate labels are denoted by ${\hat{y}}_{n,m}$. After a loss is calculated for a given minibatch, the SubNet’s trainable parameters can be fine-tuned with the help of the adaptive moment estimation (Adam) optimizer using a process known as back-propagation.

#### 3.5.3. Series and Parallel Combinations

Assume that we are considering the sum of two SubNets, A+B. Let the convolution layers in SubNet A be $L_{A}$, following Equation (5), and the convolution layers in SubNet B be $L_{B}$. $L=L_{A}+L_{B}+1$ represents the total sum of layers in the merged network, with the extra layer serving as the only completely connected one in Equation (7). SubNet A’s settings are as follows:

(14) $$\phi_{A}=\left\{w^{l},b^{l}|l\in \left\{1,2,\ldots ,L_{A}\right\}\right\}$$

This is corrected after SubNet A is trained. SubNet B’s convolution layers and the fully connected layer’s parameters are as follows:

(15) $$\phi_{B+}=\left\{w^{l},b^{l}|l\in \left\{L_{A}+1,L_{A}+2,\ldots ,L_{A}+L_{B}+1\right\}\right\}$$

As A+B is trained and the parameters in $\phi_{B+}$ are adjusted accordingly. Using the same equation as before, the output of the series layers is sent to the SoftMax layer (8).

Here, we will look at two paired SubNets, A||B. Let the convolution layers in SubNet A be $L_{A}$, following Equation (5), and the convolution layers in SubNet B be $L_{B}$. Let us set the parameters for the layer in each SubNet as

(16) $$\phi_{A}=\left\{w_{A}^{l},b_{A}^{l}|l\in \left\{1,2,\ldots ,L_{A}\right\}\right\}$$

and

(17) $$\phi_{B}=\left\{w_{B}^{l},b_{B}^{l}|l\in \left\{1,2,\ldots ,L_{B}\right\}\right\}$$

This is the result of the convolution layers in SubNet *A*:

(18) $$x_{A,n}^{l}=g\left(W_{A}^{l}x_{A,n}^{l-1}+b_{A}^{l}\right)$$

The productivity of SubNet B is assumed by the following:

(19) $$x_{B,n}^{l}=g\left(W_{B}^{l}x_{B,n}^{l-1}+b_{B}^{l}\right)$$

where $l=1,\;2,\;.\;.\;.\;,\;L_{B}$. Remember that $x_{A,n}^{0}=x_{B,n}^{0}=X_{n}$, since the inputs to the two parallel SubNets are identical. Let us call the fully interconnected output layer $L=Max\left(L_{A},\;L_{B}\right)+1$. When the final feature maps are added to the output of this completely connected layer, we obtain the following:

(20) $$x_{n}^{L}=w_{A||B}\left[\begin{array}{c} x_{A,n}^{L_{A}}\\ x_{B,n}^{L_{B}} \end{array}\right]+b_{A||B}$$

As before, the results of this layer’s work are sent to the SoftMax one via Equation (8). The parameters in $\phi_{A}$ are fixed and the parameters in $\phi_{B}$, along with $w_{A||B}$ and $b_{A||B}$, are updated.

#### 3.5.4. Proposed GraMNet Architecture

**Figure 7:**
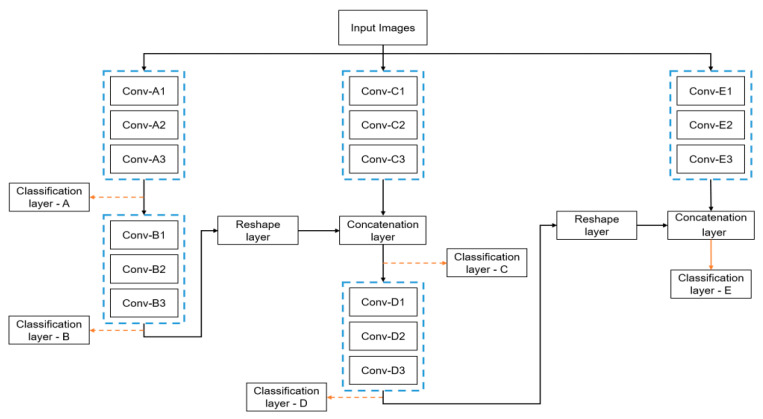
The experimental outcomes presented here were achieved using the GraMNet framework. This whole network is built from the displayed subnetworks, which are labelled A, B, C, D, and E. Table 3 details the various subnetworks.

By combining the proposed SubNets with other SubNet structures, the GraMNet can generate an infinite number of final architectures. For medical imaging, we present one exact instance that we believe strikes a good compromise between performance and computational complexity. Image 7 depicts the proposed GraMNet architecture. SubNets A, B, C, D, and E are shown in Figure 7 and are progressively added to form the whole network. Keeping the computational cost low is a primary concern, hence we employ compact and tiny SubNet modules.

To begin, we trained SubNet A using all of the available minibatches, and found the optimal parameters by minimizing the loss and employing Equation (13). Once SubNet A’s training was complete, we stopped updating its trainable parameters and instead referred to it as GraMNet A. The output of this network was the feature maps that were utilized as input by the newly created SubNet B. All the feature mappings from SubNet A’s L–1 layer were fed into SubNet B’s first convolutional layer. We called this set up GraMNet A and B, because SubNet B was connected to the rest of the network in a series arrangement that was represented by the symbol A + B.

Next, we installed a new SubNet C in tandem with locking the existing GraMNet A and B. For the sake of notational effortlessness, we referred to this set of SubNets as GraMNet A–C, or $\left(A+B\right)||C$. The equations indicated above were used to create feature maps using GraMNet A and B, which were then combined with feature maps created using the new SubNet C. With a reshape layer, we combined the depth-based feature maps from GraMNet A and B with the feature maps output from SubNet C. Finally, SubNet D was added sequentially to form the GraMNet A–D configuration $\left(A+B\right)||C+D$. At last, we parallelized GraMNet A–D and included the newest SubNet E. As a shorthand, we referred to this GraMNet series as GraMNet A–E, where $\left[\left(A+B\right)||C+D\right]||E$ is the sequence of letters. SubNets in series and parallel were chosen for this arrangement because, in our experience, they works adequately when combined and can even improve one another.

#### 3.5.5. Network Training

We used the following data partitioning to analyze the efficacy of our proposed strategy. Each class’s samples were divided as follows: 72 % were added to the training set, 8 % were added to the validation set, and 20 % were added to the test set. MATLAB’s version r2020b was used for all image dispensation and classification steps. A Windows machine sporting an Intel Xeon CPU E5-1630 v4 at 3.70 GHz was used, which sped up both the training and testing networks. We only used the training and validation datasets to train the network and fine-tune our hyperparameters for the projected GraMNet architecture. Each GraMNet was created new and given a different set of initial weights before it was trained. To cut down on the convergence time and locate the global lowest cost function for all networks, we utilized the Adam optimization method. The learning rate was scheduled to decrease by a factor of 0.1 after half of the epochs were completed. The validation frequency specified the interval in training between the validation checks. We performed validation at every 50th iteration in our settings. Taking that into account, in order to hasten the learning process and avoid over-fitting, the learning rate was kept flexible. After 0.5 epochs passed, the training rate automatically dropped by a factor of 0.1. We also utilized “Validation Patience,” which is a training policy with a value of 50. Therefore, the training procedure froze when the validation loss was more than the minimum value reached. L2 regularization [26] is a simple and effective regularization technique, and we used it with a value of 0.0001 to prevent overfitting and enhance model generalization.

#### 3.5.6. Statistical Analysis

For making comparisons between approaches, it is important to evaluate the performance of classification algorithms, choose the best one, learn about the constraints of the system, and notice areas for potential improvement [27]. 

## 4. Results and Discussion

Using Adam as an optimizer and the proposed loss function, a model was built; for testing, the conventional 5-fold cross-validation scheme was taken into account. Every fold undergoes 500 iterations of training with a batch size of 16, a learning rate of 0.0001, and a further reduction of 0.1 after 20 iterations. In the proposed work, the in-place or on-the-fly-data augmentation techniques were used [28].

### 4.1. Evaluation Metrics

In order to quantitatively assess the projected model, we employed eight widely used metrics: The Jaccard similarity index (JSI), the Dice coefficient (DC), the accuracy (ACC), the precision (PRE), the recall (REC), the specificity (SPE), the F1-score (F1), and the area under the receiver operating characteristic curve (AUC). Below, we elaborate on these measurements.

(21) $$\mathrm{JSI}=\frac{\left|A\cup B\right|}{\left|A\cap B\right|}$$

(22) $$\mathrm{DC}=\frac{2\left|A\cap B\right|}{\left|A\right|+\left|B\right|}$$

(23) $$\mathrm{ACC}=\frac{\mathrm{TP}+\mathrm{TN}}{\mathrm{TP}+\mathrm{TN}+\mathrm{FP}+\mathrm{FN}}$$

(24) $$\mathrm{PRE}=\frac{\mathrm{TP}}{\mathrm{TP}+\mathrm{FP}}$$

(25) $$\mathrm{SPE}=\frac{\mathrm{TN}}{\mathrm{TN}+\mathrm{FP}}$$

(26) $$F1=\frac{2\times \mathrm{PRE}\times \mathrm{REC}}{\mathrm{PRE}+\mathrm{REC}}$$

A and B are the real-world data and the segmentation outcomes, respectively, in Equations (21) and (22). As shown in Equations (23)–(26), the number of images that are appropriately classified as malignant is denoted by True Positives (TP), number sum of images that are properly classified as benign is denoted by True Negatives (TN), and the number of images that are wrongly classified as malignant or benign is denoted by False Positives (FP) and False Negatives (FN). The segmentation task was evaluated using JSI, DC, ACC, REC, and PRE, while the classification job was evaluated using ACC, PRE, REC, SPE, F1, and AUC.

### 4.2. Ablation Study of Proposed Model 

Table 4 represents the performance analysis of the proposed segmentation technique with the segmentation results of 3DIRCADb01. We evaluated the different experiments by considering various measures, including PRE, DC, ACC, REC and JSI. We also evaluated the five experimental results and found the following mean results: a PRE of 86.13%, a DC of 84.81%, an ACC of 88.08, a REC of 85.62% and a JSI of 84.72%.

**Figure 8:**
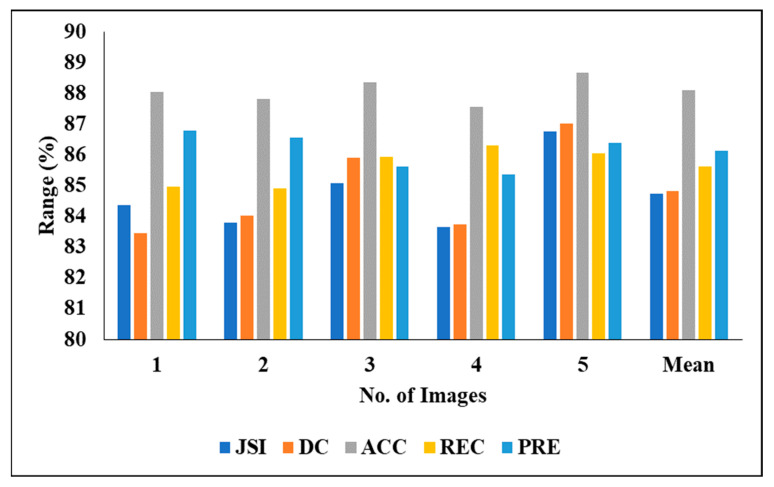
Segmentation analysis of 3DIRCADb01.

Figure 8 graphically represents the segmentation analysis of 3DIRCADb01 when considering various measures.

Table 5 represents the performance analysis of the proposed segmentation technique with the segmentation results of LITS. We evaluated the different experiment by considering various measures, including PRE, DC, ACC, REC and JSI. We also evaluated the five experimental results and found the following mean results: a PRE of 89.04%, a DC of 85.94%, an ACC of 92.17%, a REC of 87.36% and a JSI of 87.04%.

**Figure 9:**
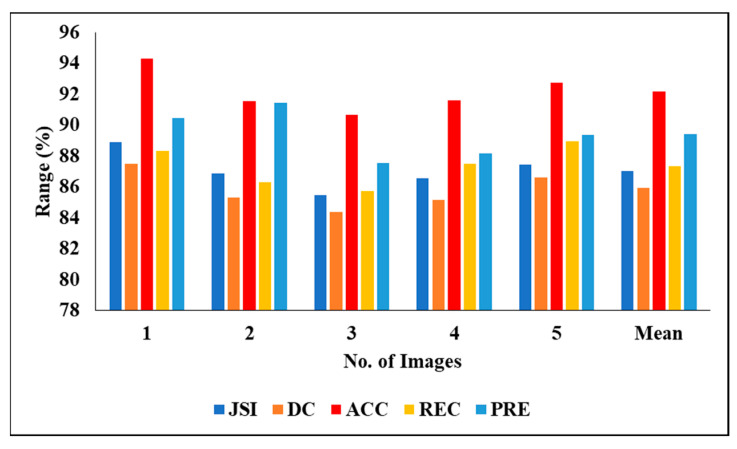
Segmentation analysis of En–DeNet on LITS.

Figure 9 graphically represents the segmentation analysis of En–DeNet on LITS when considering various measures.

### 4.3. Performance Analysis of Proposed Classification Technique

Table 6 represents the performance analysis of the proposed segmentation technique with the classification results of 3DIRCADb01. We evaluated the different experiment by considering various measures, including PRE, DC, ACC, REC and JSI. We also evaluated the five experimental results and found the following mean results: a PRE of 98.12%, an ACC of 97.86%, a REC of 98.79% and an F1 of 98.45%.

**Figure 10:**
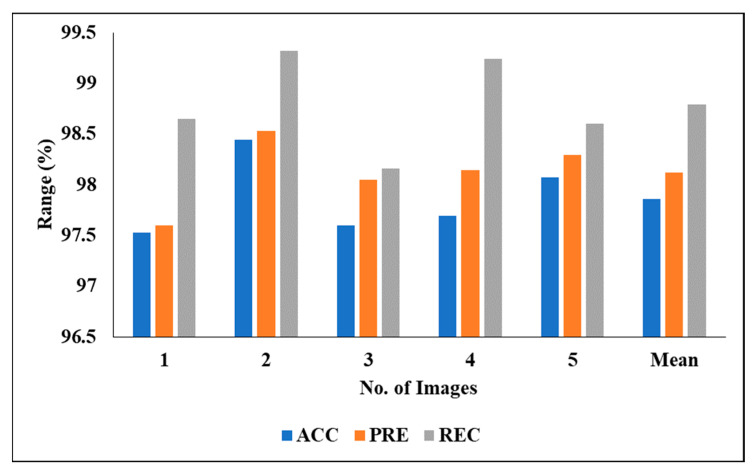
Investigation of projected classifier model on various images.

Figure 10 graphically represents the investigation of the projected classifier model when considering various images.

**Figure 11:**
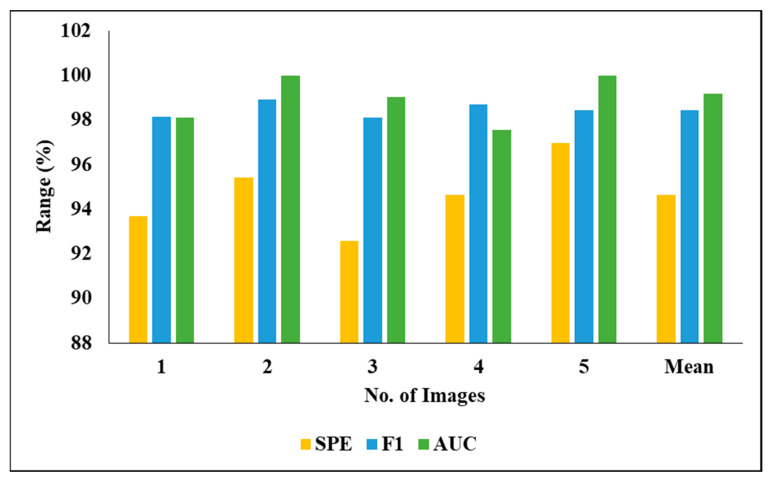
Investigation of projected classifier model on various images.

Figure 11 graphically represents the investigation of the projected classifier model when considering various images.

Table 7 represents the performance analysis of the proposed segmentation technique with classification results of LITS. We evaluated the different experiments by considering various measures, including PRE, DC, ACC, REC and JSI. We also evaluated the five experimental results and found the following mean results: a PRE of 93.65%, an ACC of 93.13%, a REC of 88.94% and an F1 of 91.42%.

**Figure 12:**
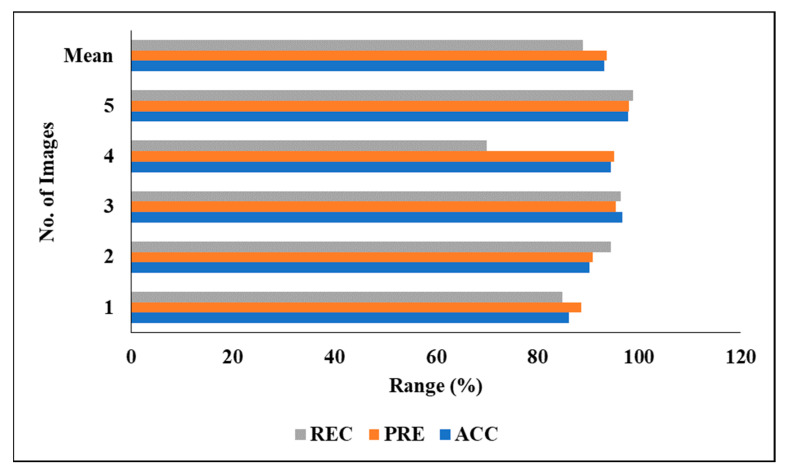
Investigation of projected classifier model on LITS.

Figure 12 graphically represents the investigation of the projected classifier model when considering LITS.

**Figure 13:**
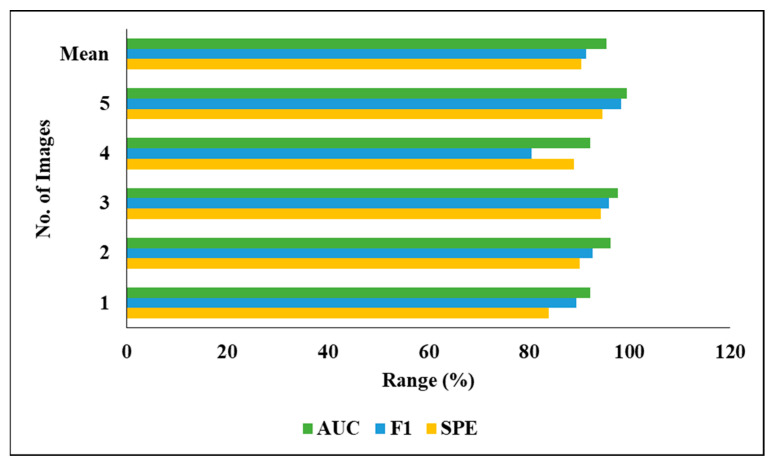
Analysis of proposed classifier model on LITS.

Figure 13 represents the analysis of the proposed classifier model on LITS in a graphical manner.

### 4.4. Comparative Investigation of Projected Perfect with Existing Procedures

The existing techniques, including HCNN [16], CFCN [18], FLAS-UNet++ [19], ECA residual UNet++ [20], GAN [22] and HCNN-CN-AEPO [21], were tested alongside our considered two datasets and the results are averaged in Table 8. The comparative investigation was carried out on 3DIRCADb01, because the mean value of the projected model was high when compared with the second dataset called LITS; therefore, only 3DIRCADb01 was considered. 

**Figure 14:**
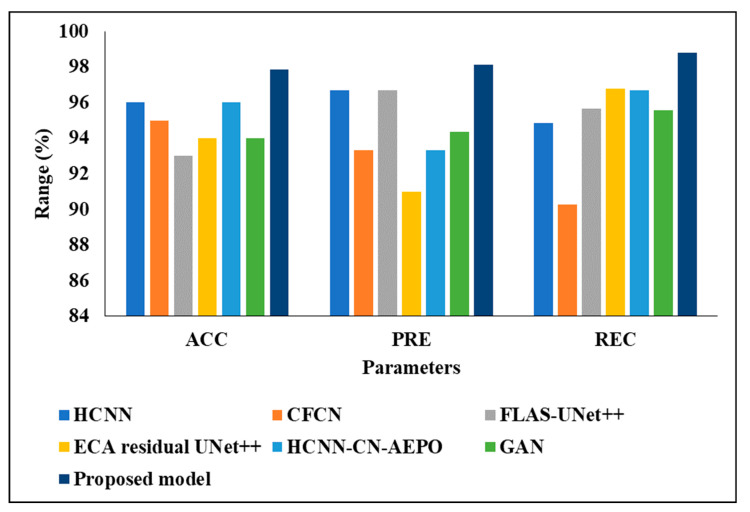
Graphical analysis of proposed GraMNet.

**Figure 15:**
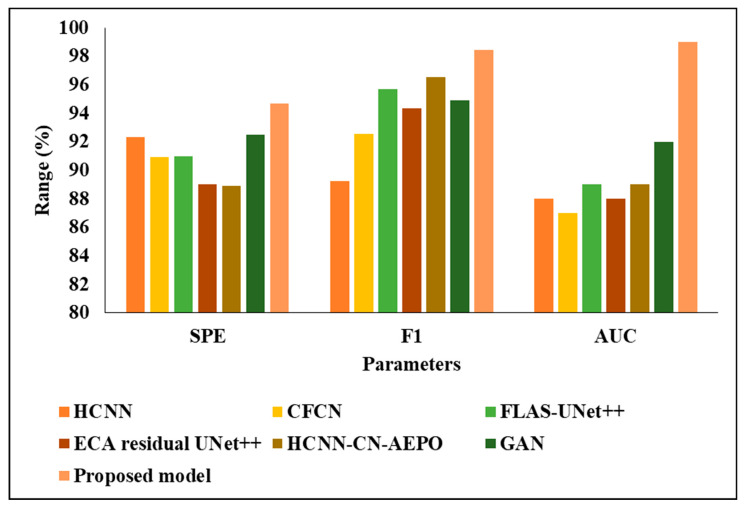
Graphical analysis of various classifiers.

In the analysis of accuracy, the existing techniques from [16,17,18,19,20,21,22] achieved an approximately 94 to 96% accuracy, where the proposed model achieved 97.86%; the reason for this better performance is that the model employed time. When the images were tested considering PRE, [20]’s technique achieved 91%, CFCN and HCNN-CN-AEPO achieved 93%, GAN achieved 94%, and HCNN and FLAS-UNet achieved 96%, but the proposed model achieved 98.12%. In addition, the proposed classifier achieved a 98% recall and F1-score, where the existing techniques from [16,17,18,19,20,21,22] achieved an approximately 92 to 96% recall and F1-score. The reasons for the low performance of the existing techniques are the high computational complexity and problems regarding a vanishing gradient when classifying the liver tumor. Figure 14 and Figure 15 present the graphical analysis of the projected model with the existing techniques in terms of various metrics.

Table 9 presents the comparative analysis of various classifiers in terms of processing time. When the training time of the input images are considered, the proposed model achieved a time of 397.33s, where the existing models achieved a training time of approximately571s to 437.17s. When the models were tested with the trained images, the results were lower. For instance, the existing models achieved a time of approximately 69s to 64s, and HCNN-CN-AEPO and GAN achieved a time of 57s to 56s. However, the proposed model achieved a testing time of 54.621s. From this analysis, it is clear that the proposed model has a reduced training and testing time compared to existing models. 

## 5. Conclusions

As part of this project, at first, the study created En–DeNet, a custom-built deep convolutional neural network model for autonomous liver image segmentation using pre-processed photos. Deep neural networks, the method perhaps most suited to application in En–DeNet, are able to detect and extract complex geometric information from liver photos. We present GraMNet, a fresh technique with which to perform deep learning model construction and training, as a result of our investigation. These networks are known as GraMNets. Via the use of two distinct imaging techniques, we have shown that the suggested strategy is effective in detecting liver cancer. For our modular method, we begin with a single SubNet and then add subsequent SubNets in either a sequential or parallel fashion. At each iteration of GraMNet, only the newest SubNet weights are modified. This method reduces the computational complexity and helps the network to learn effectively from a short training set. The overfitting problem may be overcome in many medical image analysis applications by using a monolithic deep learning approach that incorporates transfer learning.

In addition, the expansion of monolithic networks in depth and breadth results in computing costs. These problems can be overcome by the building-block GraMNet method, which trains only a single SubNet at a time and uses relatively small SubNets. According to the results section, it is clear that our GraMNet achieves results that are on par with or better than those of many well-known large-scale models. In addition, compared to the reference methods, our GraMNet trained faster, used less memory, and processed test images more quickly. We find that GraMNet has various advantages compared to monolithic deep learning from a learning perspective. Complex issues are tackled in the same modular fashion as other methods, with a number of smaller networks (called “SubNets”) being used instead of one giant one. We believe that this helps to address the vanishing gradient and other optimization issues that plague fully convolutional neural network (CNN) techniques. More importantly, our findings indicate that the GraMNet facilitates the efficient transfer of prior knowledge from the GraMNet’s fixed section to a new SubNet. There are two ways that we hope to improve GraMNet in the future by expanding its design. Firstly, the effect of integrating these SubNets in various configurations will be explored. In addition, we will examine a variety of SubNet designs.

## Figures and Tables

**Table 1:** Segmentation of liver and tumor data sets.

Dataset	Sum of Patients	Image Dimension	Pixel Width and Height	Thickness	Pixel Spacing	Slice Sum	Tumor Data out of 100%
3D-IRCADb01	20	512 × 512	0.56–0.87 mm	1–5 mm	0.55–0.95 mm	74–260	75%
LITS	131	512 × 512	–	0.7–5 mm	–	–	63%

**Table 2:** Sample of training and testing imageries.

Training Images	Testing Images
2346 + Data augmentation	392

**Table 3:** The SubNet constructions used in the GraMNet in Figure 7.

Model	Layers	Filter Size	Total Parameters	MAdd
	Conv-A1	3 × 3 × 8		
SubNet A	Conv-A2	3 × 3 × 16	0.018 M	9.94 M
	Conv-A3	3 × 3 × 32		
	Conv-B1	3 × 3 × 64		
SubNet B	Conv-B2	3× 3 × 128	0.390 M	11.94 M
	Conv-B3	3 × 3 × 256		
	Conv-C1	1 × 1 × 8		
SubNet C	Conv-C2	1 × 1 × 16	0.021 M	1.10 M
	Conv-C3	1 × 1 × 32		
	Conv-D1	3 × 3 × 64		
SubNet D	Conv-D2	3 × 3 × 128	0.390 M	43.65 M
	Conv-D3	3 × 3 × 256		
	Conv-E1	1 × 3 × 64		
SubNet E	Conv-E2	3 × 1 × 128	0.165 M	13.82 M
	Conv-E3	1 × 3 × 256		

**Table 4:** Segmentation results of 3DIRCADb01.

Exp	JSI	DC	ACC	REC	PRE
1	84.37	83.45	88.03	84.96	86.77
2	83.80	84.02	87.80	84.90	86.56
3	85.06	85.90	88.35	85.93	85.60
4	83.65	83.72	87.55	86.29	85.36
5	86.74	87.00	88.67	86.04	86.38
Mean	84.72	84.81	88.08	85.62	86.13

**Table 5:** Segmentation results of LITS.

Exp	JSI	DC	ACC	REC	PRE
1	88.91	87.51	94.32	88.32	90.45
2	86.87	85.32	91.53	86.31	91.47
3	85.44	84.35	90.67	85.74	87.56
4	86.57	85.16	91.62	87.49	88.18
5	87.45	86.63	92.75	88.95	89.35
Mean	87.04	85.94	92.17	87.36	89.4

**Table 6:** Classification results of 3DIRCADb01.

Exp	ACC	PRE	REC	SPE	F1	AUC
1	97.53	97.6	98.65	93.67	98.14	98.12
2	98.44	98.53	99.32	95.42	98.93	100
3	97.60	98.05	98.16	92.57	98.10	99.03
4	97.69	98.14	99.24	94.63	98.68	97.54
5	98.07	98.29	98.60	96.96	98.44	100
Mean	97.86	98.12	98.79	94.65	98.45	99.16

**Table 7:** Classification results of LITS.

Exp	ACC	PRE	REC	SPE	F1	AUC
1	86.30	88.65	85.00	84.00	89.43	92.14
2	90.30	91.00	94.50	90.10	92.71	96.34
3	96.70	95.41	96.45	94.33	95.92	97.71
4	94.53	95.09	70.00	89.02	80.63	92.16
5	97.86	98.12	98.79	94.65	98.45	99.52
Mean	93.13	93.65	88.94	90.42	91.42	95.38

**Table 8:** Comparison analysis on 3DIRCADb01.

Techniques	ACC	PRE	REC	SPE	F1	AUC
HCNN [16]	96.00	96.66	94.85	92.33	89.23	88
CFCN [18]	95.00	93.33	90.25	90.90	92.55	87
FLAS-UNet++ [19]	93.00	96.66	95.66	91.00	95.66	89
ECA residual UNet++ [20]	94.00	91.00	96.77	89.00	94.36	88
HCNN-CN-AEPO [21]	96.00	93.33	96.70	88.90	96.55	89
GAN [22]	94.00	94.33	95.55	92.47	94.91	92
Proposed model	97.86	98.12	98.79	94.65	98.45	99

**Table 9:** Processing time of proposed model.

Model	Training Time (s)	Testing Time (s)
HCNN [16]	452.53	69.38
CFCN [18]	556.21	68.89
FLAS-UNet++ [19]	567.66	66.71
ECA Residual UNet ++ [20]	490.23	64.41
HCNN-CN-AEPO [21]	571.42	57.78
GAN [22]	437.17	56.192
Proposed Model	397.33	54.621

## Data Availability

The datasets used and/or analyzed during the current study are available from the corresponding author on reasonable request.
